# Neurobiology of Anorexia Nervosa: Serotonin Dysfunctions Link Self-Starvation with Body Image Disturbances through an Impaired Body Memory

**DOI:** 10.3389/fnhum.2016.00600

**Published:** 2016-11-24

**Authors:** Giuseppe Riva

**Affiliations:** ^1^Applied Technology for Neuro-Psychology Lab, Istituto Auxologico ItalianoMilan, Italy; ^2^Centro Studi e Ricerche di Psicologia della Comunicazione, Università Cattolica del Sacro CuoreMilano, Italy

**Keywords:** 5-HTTLPR, serotonin transporter gene, serotonin, anorexia nervosa (AN), allocentric lock, body image disturbances, memory consolidation, memory reconsolidation

## Abstract

The etiology of anorexia nervosa (AN) is still unclear, despite that it is a critical and potentially mortal illness. A recent neurobiological model considers AN as the outcome of dysfunctions in the neuronal processes related to appetite and emotionality (Kaye et al., 2009, 2013). However, this model still is not able to answer a critical question: What is behind body image disturbances (BIDs) in AN? The article starts its analysis from reviewing some of the studies exploring the effects of the serotonin systems in memory (episodic, working, and spatial) and its dysfunctions. The review suggests that serotonin disturbances may: (a) facilitate the encoding of third person (allocentric) episodic memories; (b) facilitate the consolidation of emotional episodic memories (e.g., teasing), if preceded by repeated stress; (c) reduce voluntary inhibition of mnestic contents; (d) impair allocentric spatial memory. If we discuss these results within the interpretative frame suggested by the “Allocentric Lock Hypothesis” (Riva, 2012, 2014), we can hypothesize that altered serotoninergic activity in AN patients: (i) improves their ability to store and consolidate negative autobiographical memories, including those of their body, in allocentric perspective; (ii) impairs their ability to trigger voluntary inhibition of the previously stored negative memory of the body; (iii) impairs their capacity to retrieve/update allocentric information. Taken together, these points suggest a possible link between serotonin dysfunctions, memory impairments and BIDs: the impossibility of updating a disturbed body memory using real time experiential data—I'm locked to a wrong body stored in long term memory—pushes AN patients to control body weight and shape even when underweight.

## Introduction

Although anorexia nervosa (AN) is a critical and potentially mortal illness (Mustelin et al., 2016), its etiology is still unclear (Kaye et al., 2013). Recent neurobiological studies consider AN as the outcome of dysfunctions in the neuronal processes related to appetite and emotionality (Kaye et al., 2009, 2013).

In their reviews, Kaye et al. (2009, 2013) suggest that AN patients are characterized by a dysregulation in the anterior ventral striatal pathway that may create a vulnerability for dysregulated appetitive behaviors. The high level of self-control in individuals with AN—produced by an exaggerated dorsal cognitive circuit functioning—allows them to inhibit appetite.

This model, even if very influential and able to provide clear suggestions for therapy, still is not able to answer a critical question: What is behind body image distortion in AN? As noted by Kaye et al. (2013): “This may be the most puzzling of all AN symptoms, in part because AN individuals feel fat but tend to have normal perceptions of other people's bodies” (p. 117).

A possible path for providing an answer to this question is to investigate the role of altered monoamine neural modulation in AN (Haleem, 2012).

The term “monoamine neurotransmitters” refers to the particular dopamine (DA), noradrenaline (NAD), adrenaline (AD), and serotonin (5-HT) neurotransmitters that are released from neurons in both the brain and peripheral nervous system (Kaye et al., 1984) and that affect a variety of psychobiological factors including hunger, anxiety, impulsivity, perception, and memory. These neurotransmitters have been extensively investigated, and different studies suggest a significant decrease in AN patients when compared to normal subjects (Kaye et al., 2013). In particular, the 5-HT and DA systems have a significant impairment in AN patients (Kaye et al., 2009), with possible effects on satiety, impulse control, and mood (5-HT), and aberrant rewarding effects (DA) of motivation and food (Kaye et al., 2013).

Although the possible role of dopamine in AN is still controversial (O'Hara et al., 2015; Peng et al., 2016; Södersten et al., 2016), a number of studies evidenced an altered serotoninergic activity in AN (Kumar et al., 2010; Calati et al., 2011; Jean et al., 2012; Chen et al., 2015) and demonstrated the role of the 5-HT receptors located in the hypothalamus in food intake and body weight control (Compan et al., 2012; Haleem, 2012). In this view, the 5-HT impairment may have a clinical role in explaining the insufficient food consumption in AN. As underlined by Compan et al. (2012): “The brain 5-HT system is central in the control of food intake and particularly in eating disorders…Accordingly, environmental changes (stress) could alter the adaptive decision-making concerning feeding. If the adaptive response to stress depends on the 5-HT system, eating disorders could thus emerge when 5-HT neurons reach the limit of their adaptive capacities.” (p. 723). Additionally, early-life stress, a risk factor for eating disorders (Su et al., 2016), induces persistent changes in 5-HT receptors and transporter (Bravo et al., 2014).

However, recent studies also suggest a link between 5-HT and memory dysfunctions, creating a possible bridge between serotonin disturbances, impaired body memory, and body image disturbances.

**Figure 1:**
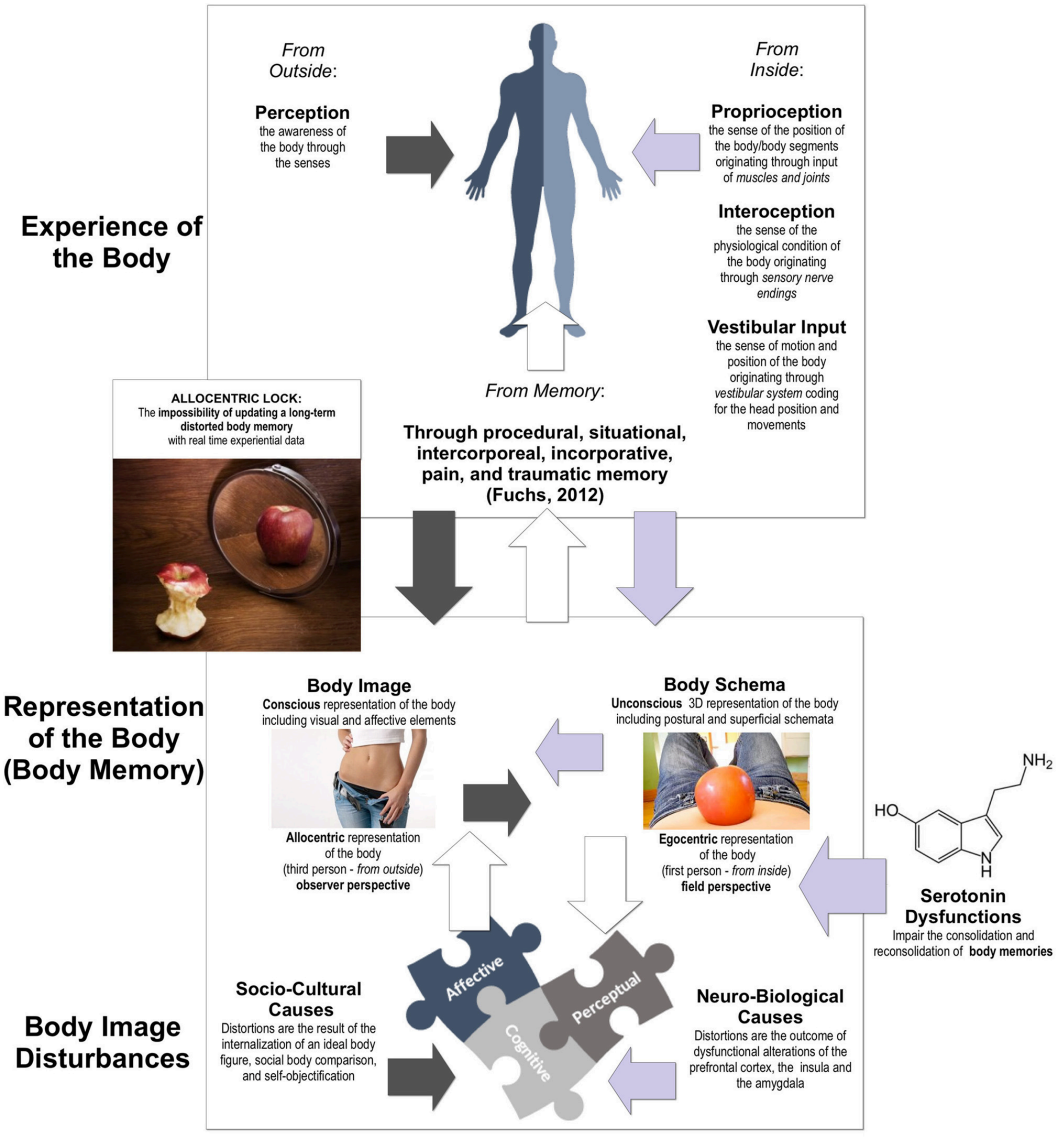
**Body Image Disturbances and their link with the Allocentric Lock Hypothesis**.

Our experience of the body is not direct (Figure 1), but it is mediated by perceptual information, recalibrated through stored information (body representations) and influenced by internal information—proprioception, interoception, and vestibular input (Blanke et al., 2015; Pazzaglia and Zantedeschi, 2016).

In this view, body image distortion can be seen as a multidimensional construct (Figure 1) that, according to recent neuroimaging studies, includes three different components: affective, cognitive, and perceptive, (Gaudio and Quattrocchi, 2012). The affective and cognitive components of body image distortion are widely accepted and related both to socio-cultural issues (van den Berg et al., 2002; Tylka, 2011; Swami, 2015)—internalization of an ideal body figure (Fitzsimmons-Craft et al., 2016), social body comparison (Andrew et al., 2016), and self-objectification (Dakanalis et al., 2016a)—and/or to brain dysfunctions (Sato et al., 2013; Suchan et al., 2015; Esposito et al., 2016)—alterations of the prefrontal cortex, the insula and the amygdala (Dakanalis et al., 2016b).

The interest for the perceptual component of body image distortion is more recent and related to the outcomes of different functional magnetic resonance imaging (fMRI; Mohr et al., 2010; Suchan et al., 2013). Gaudio and colleagues summarizing these studies in a recent systematic review (Gaudio et al., 2016) conclude “that several brain regions could be involved in body image disturbances and may sustain an impaired integration between real and perceived internal/external state of one's own body in AN patients” (p. 582). For example, neuroimaging studies have demonstrated both functional and structural more marked alterations in visual areas, with anomalies in body-image processing related to self but not to others (Uher et al., 2005; Sachdev et al., 2008). A specific association between therapy-related changes and modulation of the BOLD signal in these areas suggests that this type of distortion of the body's configuration is based on visual contributions (Vocks et al., 2011). Specifically, a recent study showed that AN patients' perception of their own body is more easily malleable by exposure to round figures as compared to controls (Cazzato et al., 2016). Nevertheless, another systematic review underlined a multisensory impairment of body perception in AN that goes beyond visual misperception and involves tactile and proprioceptive sensory components and suggests a critical role of memory in these processes (Gaudio et al., 2014). As suggested by two recent reviews (Longo, 2015; Martijn et al., 2015) and underlined by Fuchs (2012): “The body is not only a structure of limbs and organs, of sensations and movements. It is a historically formed body whose experiences have left their traces in its invisible dispositions…Sensations or situations experienced by the lived body may function as implicit memory cores which, under suitable circumstances, can release their enclosed memories.” (p. 20).

An emerging etiological model of AN, that focuses on body memories is the “*Allocentric Lock (AL) Hypothesis*” (Riva, 2012, 2014; Riva et al., 2015).

This theory suggests, that AN is the outcome of a primary disturbance in the way the body is “experienced” and “remembered.” Specifically, individuals with (or developing) this disorder may be locked to an allocentric (third person) disturbed memory of their body that, independently of its causes, is not more updated by experiential data, even after a successful diet and/or a significant weight reduction (Figure 1—for a broader review see: Riva, 2007, 2011, 2012, 2014; Gaudio and Riva, 2013; Riva et al., 2015; Dakanalis et al., 2016b).

The article highlights some of the important studies discussing the effects of 5-HT markers (i.e., receptors and transporter) on memory dysfunctions, which may play a role in clarifying the link between decreased serotonin transmission, body memory, and body image disturbances.

## The role of 5-HT in working, spatial, and episodic memory

A wealth of experimental animal studies demonstrate the role of the serotonin systems in memory and its dysfunctions, even if their role is still poorly understood (Meneses et al., 2011; Meneses, 2013; Gasbarri and Pompili, 2014; Gasbarri et al., 2016; Meneses and Gasbarri, 2016). It is beyond the scope of this paper to describe the results of all these studies (for an in-depth analysis please refer to Meneses et al., 2011; Roberts and Hedlund, 2012; Meneses, 2013; Glikmann-Johnston et al., 2015; Meneses and Gasbarri, 2016). In this context, we will focus only on the studies more relevant to our discussion.

### 5-HT receptors and memory

The serotonin receptors, activated by the neurotransmitter serotonin, mediate both excitatory, and inhibitory neurotransmission in the central and peripheral nervous systems. They are classified in seven main receptor subtypes: 5-HT_1−7_. Also, 5-HT_1_ receptors include 5-HT_1A_, 5-HT_1B_, and 5-HT_1D_ subtypes; while 5-HT2 receptors include 5-HT_2A_, 5-HT_2B_, and 5-HT_2C_ subtypes.

#### Episodic memory

The role of 5-HT receptors in episodic memory was recently explored in animal studies by Zhang et al. (2013, 2016), Bekinschtein et al. (2013), and Morici et al. (2015). On one side, the activation of 5-HT_2A_ receptor improved object memory consolidation, without affecting encoding or retrieval, (Zhang et al., 2013, 2016) and enhanced the consolidation of contextual and cued fear memories (Zhang et al., 2013).

On the other side, the loss of 5-HT_2a_ receptors produced deficits in the ability to remember both an association between the objects and the context in which they were seen (object-in-place associations; Bekinschtein et al., 2013; Morici et al., 2015), and the objects and their relative position in time (Morici et al., 2015). However, this deficit was not general but it related to the level of interference (Morici et al., 2015): the deficit appeared when the interference level was high, suggesting a role for the 5-HT_2*a*_ receptor in memory interference resolution. Interestingly, an impairment in memory interference resolution is also associated with alexithymia (Coligan and Koven, 2015). Another factor influencing the role of 5-HT in episodic memory is stress. A recent study showed that serotonergic fear memory consolidation in rats, induced by an infusion of a 5-HT_2*C*_ receptor antagonist, happened only after a history of repeated stress exposure (Baratta et al., 2016).

In agreement with this and other results (Ballaz et al., 2007; Ohmura et al., 2015), both the use of serotonergic reuptake inhibitors (SSRIs) and serotonergic–noradrenergic reuptake inhibitors (SNRIs) in a human study significantly improved the episodic memory and to a lesser extent, working memory (Herrera-Guzmán et al., 2009). This finding is in line with a study by Mlinar et al. (2015) showing that in rats, hippocampal long-term potentiation at CA3/CA1 synapses was facilitated by endogenous 5-HT.

#### Working memory

In a first animal study, Zhang and colleagues explored the effects of the activation of 5-HT_2A_ receptors in rats (Li et al., 2015). Their data underlined an enhancement of working memory (increased choice accuracy in the T-maze rewarded alternation test) after the injection of the 5-HT_2A_ receptor agonist. A similar result was reported by López-Vázquez et al. (2014).

In another animal study, Gonzalez-Burgos et al. (2012) explored the effects of prefrontal serotonin depletion on the memory strategies (allocentric and egocentric) used in a working memory task. The results suggested that serotonin may be involved in the prefrontal organization of egocentric working memory, based on own movement-guided responses.

#### Spatial memory

In an animal study, Gutiérrez-Guzmán et al. (2011) produced 5-HT hippocampal depletion through lesions to the cingulate bundle, fimbria, and fornix of rats. The hippocampal 5-HT depletion facilitated place learning accuracy. In a second study, the same authors (Gutiérrez-Guzmán et al., 2012) lesioned serotonergic terminals of the supramammillary/posterior hypothalamus nuclei in rats. Their data suggested a significant role of 5-HT in the intermediate- and long-term consolidation of spatial information (Gutiérrez-Guzmán et al., 2012). In particular, different animal studies, using 5-HT_7_ receptor knockout mice, showed an impairment in the recognition of novel locations but not in the recognition of novel objects (Ballaz et al., 2007; Sarkisyan and Hedlund, 2009). A similar result was found in different studies involving activation or blockade of the 5-HT_1A_: if higher levels of 5-HT maintained or improved spatial memory, reduced levels of 5-HT impaired spatial memory (Glikmann-Johnston et al., 2015).

A possible explanation for these data comes from a computational network model used to investigate 5-HT modulation on spatial working memory (Cano-Colino et al., 2014). Its results suggest that serotonin modulates spatial working memory performance nonmonotonically via 5-HT_1A_ (Koenig et al., 2008) and 5-HT_2A_ (Bekinschtein et al., 2013) receptors.

### 5-HT transporter and memory

The serotonin transporter (SERT) is an integral membrane protein with the role of taking up serotonin released during serotonergic neurotransmission by transporting it from synaptic spaces into presynaptic neurons (Meneses et al., 2011; Coleman et al., 2016). Numerous gene variants have been identified, which have a significant impact on its functioning. The most studied of these SERT gene variants is the SERT gene-linked polymorphic region (5-HTTLPR), which results in a short or long form (Nakamura et al., 2000; Segal et al., 2009): the short form is characterized by a reduction in SERT mRNA, SERT binding, and 5-HT when compared with the long form.

#### Episodic memory

Olivier et al. (2009) in a study using different SERT knock-out rats, found that SERT −/− and SERT +/− rats showed evidence of impaired object memory. The impairment was not found in SERT +/+ rats.

Wu and colleagues recently evaluated the effects of SERT gene knockdown on contextual fear memory in mice (Wu et al., 2016). Their results, in agreement with previous studies (Dai et al., 2008; Sivamaruthi et al., 2015), suggested that SERT knockdown impairs the extinction of contextual fear memory (Wu et al., 2016). Line and colleagues, in another animal study of mice over-expressing the SERT (Line et al., 2014), found an impairment on appetitive and aversively motivated learning tasks, suggesting a role for serotonin in the processing of both aversive and rewarding stimuli (McCabe et al., 2010).

In a human study, Lemogne et al. (2009) found that the 5-HTTLPR polymorphism moderated the effects of life stress on visual perspective for positive memories: individuals with at least one low or long _G_ allele used an allocentric perspective for positive memories during life stress more than individuals did who were homozygous for the long _A_ allele.

#### Working memory

A study involving both cocaine users and controls genotyped for 5-HTTLPR polymorphisms underlined a significant gene × environment interaction related to the role of serotonin in working memory (Havranek et al., 2015). In cocaine users, 5-HTTLPR long genotype was a risk allele for a worse working memory performance, whereas in healthy controls, it was associated with better working memory performance. Analogously, high SERT mRNA levels were associated with working memory impairments in cocaine users, but with increased performance in normal subjects. A link between working memory and 5-HTTLPR polymorphisms was also found by Konrad et al. (2011) and by Price et al. (2013): female normal subjects with the 5-HTTLPR low allele evidenced a poorer working memory performance.

#### Spatial memory

In their review, Kalueff et al. (2010) discussed the spatial memory performance of SERT gene knockdown (−/−) mice and rats. In their view (Kalueff et al., 2010), “The absence of the SERT slightly impairs hippocampus-dependent spatial/object memory, in striking contrast with improved amygdala-dependent emotional memory (e.g., fear conditioning) in SERT (−/−) rodents.” (p. 382).

## The role of 5-HT systems in the allocentric lock

**Figure 2:**
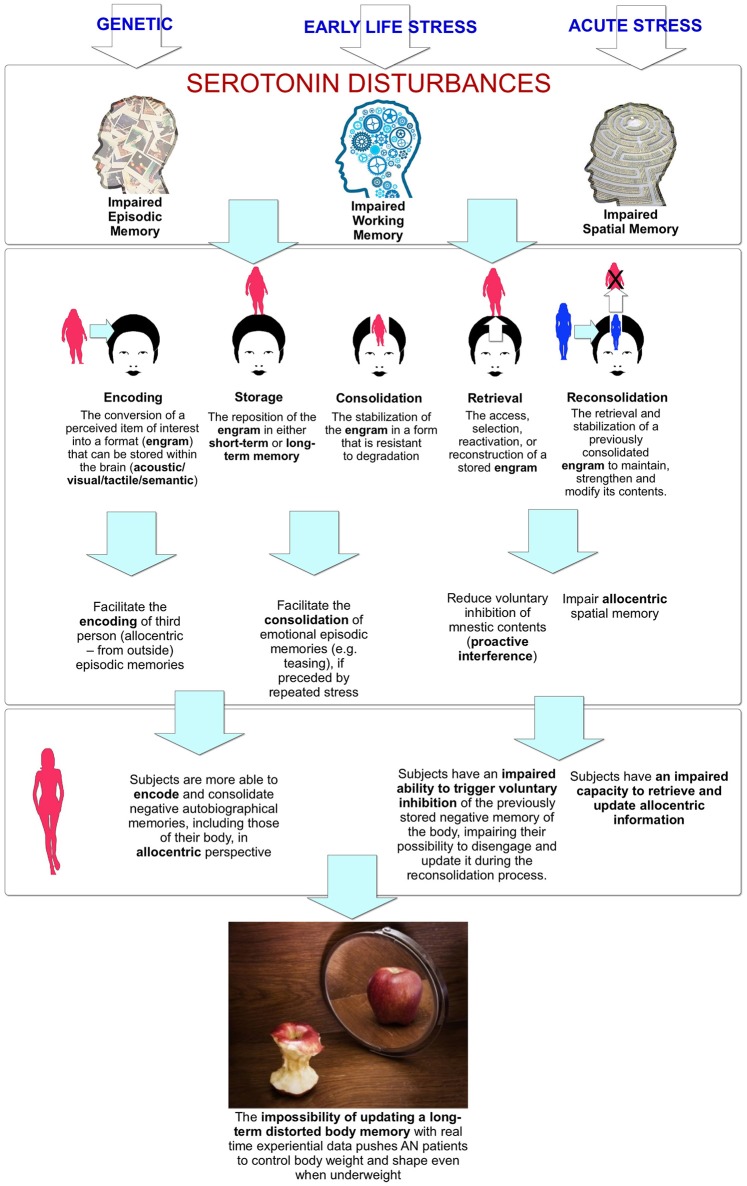
**The link between serotonin disturbances and body memory impairments**.

The studies discussed above, even if largely based on animal research, suggest a link between the 5-HT systems and the memory processes—encoding/storage, consolidation, and retrieval/reconsolidation (see Figure 2)—involved in the allocentric lock. Furthermore, 5-HT effects appear to be modulated by stress (Baratta et al., 2016) and estrogen (Epperson et al., 2012), and have a stronger influence on working and short-term memory than on long-term memory (Hritcua et al., 2007).

In relation to encoding and storage, the study by Lemogne et al. (2009) suggested that the 5-HTTLPR polymorphism influences the visual perspective used for storing positive memories during life stress. Specifically, individuals with at least one low or long _G_ allele used more a third person perspective in episodic memory, facilitating the storing of an allocentric memory of their body. More, the results of the study by Gonzalez-Burgos et al. (2012) showed that serotonin depletion impaired the egocentric working memory performance while no effects were found for the allocentric one.

In relation to the consolidation process, it is well known that in humans, it is impaired by a decrease in serotonergic neurotransmission (Sivamaruthi et al., 2015). Animal studies also have found that a decrease in 5-HT reduced conditioned responses in both short and long-term memory (Gonzalez et al., 2013), while its availability improved the stabilization of emotional memories (Baratta et al., 2016), if preceded by a history of repeated stress exposure (Ohmura et al., 2015).

The reviewed studies also suggested a critical role of 5-HT in memory retrieval and reconsolidation. In humans, serotonin receptor *5-HTR*_2A_ gene polymorphism was associated with a significant impairment in memory retrieval (de Quervain et al., 2003; Sigmund et al., 2008). Also, if we focus on the computational network model used to investigate 5-HT modulation on spatial working memory (Cano-Colino et al., 2014), its results emphasized a critical role of 5-HT in the inhibition of currently irrelevant or unwanted information (Marsh et al., 2012). According to the authors: “Increasing levels of tonic [5-HT] …favored spatial working memory by suppressing unwanted false memories” (p. 2459). Sarkisyan and Hedlund, discussing the results of their study (Sarkisyan and Hedlund, 2009), suggested: “The 5-HT_7_ receptor might be important in the formation of associations with the network of memories and the compilation and correlation of these memories with changes in the environment” (p. 29).

Recalling information from long-term memory usually requires the selection of a specific target from a wider set of targets competing for access. Taken together, these effects suggest a role of serotonin in inhibiting unwanted targets (proactive interference) that can be impaired during decreased serotonin transmission. Two studies with monkeys confirmed this interpretation (Clarke et al., 2004, 2007): selective serotonin depletion of the orbitofrontal cortex impairs the ability to switch between visual stimuli when responding on a serial discrimination reversal task. Specifically, as the authors noted: “The failure of 5-HT-lesioned monkeys to cease responding to the previously correct stimulus was due to an inability to disengage from that stimulus” (p. 24). Furthermore, a recent study also points out the role of serotonin in memory reconsolidation (Nikitin et al., 2016): in snails, the 5-HT receptor antagonist induced the disruption of memory reconsolidation related to previously conditioned food aversion.

Finally, if we focus on the brain systems involved in the allocentric computations (Ekstrom et al., 2014)—*hippocampus, retrosplenial cortex*, and *parahippocampal cortex*—we can find a significant serotonergic innervation affecting the proliferation and activity of their cells.

For example, in mice, 5-HT receptors induce spine growth in the CA1 (Restivo et al., 2008), an area of the hippocampus involved in spatial autobiographical memory and in the development of allocentric view independent representations. It is also well-known that serotonin synthesis has a positive regulatory factor on the granule cell layers of the retrosplenial cortex, a brain area involved in transforming allocentric representations into egocentric ones (Vann and Aggleton, 2005; Vann et al., 2009) by improving their proliferation (Richter-Levin and Segal, 1990; Brezun and Daszuta, 2000). A decreased serotonin transmission may impair these areas, disrupting their functions. A study of 5-HT7 receptor-deficient mice (5-HT7 –/–) supported this hypothesis (Sarkisyan and Hedlund, 2009): in a spatial memory task the mice demonstrated an impaired allocentric spatial memory whereas egocentric spatial memory remained intact. Also, different recent neuroimaging studies with AN patients revealed significant brain dysfunctions in the key areas involved in the allocentric computation (Riva and Gaudio, 2012; Gaudio and Riva, 2013).

## Conclusions

In conclusion, this review suggests that on one side, 5-HT systems modulate memory and its dysfunctions; on the other side, a decreased serotonin level impairs the different memory processes—encoding/storage, consolidation, and retrieval/reconsolidation—involved in episodic and autobiographical memory (see Figure 2). Specifically, serotonin disturbances:

- Facilitate the encoding of allocentric (from outside) episodic memories;
- Facilitate the consolidation of emotional episodic memories (e.g., teasing), if preceded by repeated stress;
- Reduce voluntary inhibition of mnestic contents;
- Impair allocentric spatial memory.

If we discuss these data within the interpretative frame suggested by the Allocentric Lock Hypothesis, we can hypothesize that AN patients:

- Are more able to store and consolidate negative autobiographical memories, including those of their body, in allocentric perspective. As demonstrated by Eich et al. (2009, 2012), these memories produce a significant reduction in one's cortical representations of the physical self. As clarified by the authors (Eich et al., 2012): “When we choose to relive past events from a perspective outside our body, we shut down the neural circuitry in the insula that is central for monitoring our bodies' internal states” (p. 177). A first support for this hypothesis is given by the multisensory impairment of body perception existing in AN patients, suggesting that their bodily experience is shaped by sensorimotor/proprioceptive memory (Gaudio et al., 2014).
- Have an impaired ability to trigger voluntary inhibition of the previously stored negative memory of the body, impairing their possibility to disengage, and update it during the reconsolidation process. Two recent studies with AN patients provide a preliminary support for this vision. The first study, involving both patients with anorexia and normal subjects genotyped for 5-HTTLPR polymorphisms (Collantoni et al., 2016), provided evidence of an impaired response inhibition in AN patients and suggested an important role of the serotoninergic system in inhibitory control (Conway and Fthenaki, 2003). A second study by Bomba et al. (2014), with a sample of both AN and healthy volunteers, showed an overgeneralization of autobiographical memory in AN patients, usually explained by the difficulty in ignoring interference from irrelevant cognitions (Smets et al., 2014).
- Have an impaired capacity to retrieve and update allocentric information. A recent study by Serino et al. (2016) offered a first support for this hypothesis. The study showed that both AN and bulimic patients were significantly less accurate in retrieving and updating—within an allocentric frame of reference—the position of an object previously memorized using an egocentric viewpoint.

In conclusion, the data accumulated in this review suggest a possible link between serotonin dysfunctions and body image disturbances in AN: the impossibility of updating a disturbed body memory using real time experiential data—I'm locked to a wrong body stored in long term memory—pushes AN patients to control body weight and shape even when underweight. This view is in agreement with a recent hypothesis that describe anorexia as a disturbance of the self (Amianto et al., 2016) specifically associated “with spatial functioning possibly related to experiencing one's own body as an integrated aspect of the self, and temporal functioning possibly related to integrating the self in a coherent narrative over time.” (p. 7). More, it is also in agreement with the free energy framework suggesting that pre-existing mental models shape current perception (Friston and Kiebel, 2009). According to this vision the brain maintains hypotheses of the causes of sensory input predicting inputs, which are then compared with actual sensory input. In this view body image disturbances may be the outcome of dysfunctional predictive mechanisms (Seth et al., 2012; Di Lernia et al., 2016).

However, it is also true that this review is largely based on animal studies. Due to this, there exists a theoretical gap between the outcome of these studies and the possible effects of serotonin in humans. Further, studies are needed to explore and clarify the link between human serotonin dysfunctions, body image disturbances, and the causes of AN.

## Author contributions

GR conceived, collected, analyzed, interpreted the data, and gave his final approval of the version to be published.

## Funding

This paper was supported by the PRIN 2015 project “Unlocking the memory of the body: Virtual Reality in Anorexia Nervosa” (201597WTTM).

### Conflict of interest statement

The author declares that the research was conducted in the absence of any commercial or financial relationships that could be construed as a potential conflict of interest. The reviewer EW and handling Editor declared their shared affiliation, and the handling Editor states that the process nevertheless met the standards of a fair and objective review.
